# Efficacy of Sofosbuvir–Velpatasvir–Ribavirin Regimen for Retreatment of Chronic Hepatitis C in Patients With Prior DAA Failure: A Retrospective Study From a National Treatment Center

**DOI:** 10.1155/ijh/9918357

**Published:** 2025-09-04

**Authors:** Sinku Singh, Sibangi Sengupta, Deepak Kumar, Kousik Mukherjee, Abdulla M. D. Hasan, Provash Sadhukhan, Sk Mahiuddin Ahammed

**Affiliations:** ^1^Department of Gastroenterology, Institute of Postgraduate Medical Education and Research, Kolkata, West Bengal, India; ^2^Department of Hepatology, Institute of Postgraduate Medical Education and Research, Kolkata, West Bengal, India; ^3^ICMR-National Institute for Research in Bacterial Infections, Kolkata, West Bengal, India

**Keywords:** DAA, HCV retreatment, HCV treatment failure, National Viral Hepatitis Control Program, sofosbuvir, velpatasvir

## Abstract

**Introduction:** Direct-acting antivirals (DAAs) are highly effective in treating HCV infection, but a small subset of patients may fail to achieve SVR12 and require further intervention. In resource-limited settings where second-line DAAs (such as SOF/VEL/VOX) may be unavailable, optimizing first-line treatments is essential. This study evaluated the efficacy (SVR12) of a retreatment regimen based on first-line DAAs (SOF/VEL) with ribavirin.

**Method:** This retrospective study screened all viremic patients who attended the apex treatment center (ATC) between January 2019 and December 2023 and received DAAs as per the National Viral Hepatitis Control Program (NVHCP) guidelines. Patients who failed to achieve SVR12 were subsequently retreated with the available first-line regimen (SOF/VEL plus ribavirin).

**Results:** A total of 1814 viremic patients attended the ATC. One thousand two hundred and sixty-two patients completed therapy. One thousand one hundred ninety-eight (94.9%) patients achieved SVR12, and 64 patients (5.1%) failed to achieve SVR12. Additionally, 41 patients with DAA failure were referred from the treatment center (TC) and model treatment center (MTC) for evaluation. After further exclusions, 36 patients were enrolled, and 30 of them were offered retreatment. The majority of patients were male (64.5%) with a median age of 45 years (IQR, 19–68). Five patients were cirrhotic, while the remainder was noncirrhotic. Baseline HCV RNA levels before the retreatment regimen were 87,882 IU/mL (IQR, 9870–484,902). Most patients (96.6%) had Genotype 3 HCV infection. Prior to retreatment, 27 patients had received a 12-week regimen of sofosbuvir and daclatasvir, while only three had been treated with the sofosbuvir–velpatasvir regimen. After retreatment with sofosbuvir, velpatasvir, and ribavirin, 22 patients (73%) achieved SVR12. None of the patients experienced any adverse event.

**Conclusion:** First-line DAAs are highly effective to treat naïve patients. In the absence of second-line options, retreatment with first-line DAAs (SOF/VEL plus ribavirin) is a viable alternative.

## 1. Introduction

Hepatitis C virus (HCV) infection contributes significantly to global morbidity and mortality, with an estimated 75 million people living with chronic HCV infection worldwide, according to the World Health Organization (WHO) [1]. In India, HCV prevalence is estimated at 0.3%–0.5%, translating to a substantial number of infected individuals due to the country's large population [2].

The WHO has set ambitious targets to eliminate viral hepatitis as a public health threat by 2030, aiming to diagnose 90% of chronic hepatitis cases and treat 80% of HCV patients [3]. Currently, however, only 9%–20% of patients are aware of their virological status, and only a fraction of those affected are receiving treatment, underscoring an urgent need to improve access and ensure treatment for more individuals with chronic viral hepatitis.

In response, the government of India launched the National Viral Hepatitis Control Program (NVHCP), which seeks to scale up comprehensive screening, linkage to care, and treatment [4]. The program provides access to highly effective pangenotypic drugs, including sofosbuvir–daclatasvir (SOF/DCV) and sofosbuvir–velpatasvir (SOF/VEL). According to NVHCP guidelines, a 12-week course of SOF/DCV is recommended for patients with noncirrhotic HCV. A 12-week course of SOF/VEL is recommended for patients with compensated cirrhosis. For patients with decompensated cirrhosis, a 24-week course of SOF/VEL or a 12-week course of sofosbuvir, velpatasvir, and ribavirin (SOF/VEL/RBV) is recommended [4].

Direct-acting antiviral (DAA) regimens generally demonstrate high efficacy, with cure rates between 90% and 98% [5, 6]. However, some factors—such as Genotype 3 and cirrhosis—may reduce response rates [7]. In India, where Genotype 3 is predominant, NVHCP does not recommend genotyping, applying a uniform treatment regimen for all patients. This approach could potentially lead to lower efficacy rates. Furthermore, with NVHCP's focus on broad screening, linkage to care, and decentralized treatment centers (TCs), a significant number of patients in real-world settings may not achieve a cure and may require retreatment.

However, a defined retreatment regimen is currently lacking in NVHCP. International guidelines, such as those from the European Association for the Study of the Liver (EASL) and the American Association for the Study of Liver Diseases (AASLD), recommend the sofosbuvir–velpatasvir–voxilaprevir (SOF/VEL/VOX) combination as the retreatment option of choice for patients who have not responded to previous DAA regimens [8, 9]. Unfortunately, this regimen is not available within NVHCP. There is limited data regarding the efficacy of SOF/VEL with ribavirin as a retreatment regimen. A study from India by Goel et al. used SOF/VEL/RBV as a retreatment regimen and achieved a sustained virological response (SVR) rate of 75% [10]. Therefore, in our study, we aimed to assess the outcomes of a retreatment regimen based on available drugs, specifically SOF/VEL/RBV (SVR regimen).

## 2. Materials and Methods

### 2.1. Study Design, Approvals, and Participants

This retrospective study was conducted in the outpatient department (OPD) of the NVHCP at the apex treatment center (ATC), IPGME&R, Kolkata, West Bengal. The study protocol was adhered to the Declaration of Helsinki and received ethical clearance from the IPGME&R Research Oversight Committee, vide Memo No. IPGME&R/IEC/2024/0015. Written informed consent was obtained from patients who required a retreatment regimen.

The study screened all HCV viremic patients who attended the NVHCP OPD from January 2019 to December 2023. After screening, the study enrolled adults (age > 18 years) who have received first-line DAA (SOF/DCV or SOF/VEL) regimen under the NVHCP program and failed to achieve sustained virological response at 12 weeks (SVR12). In addition to our center, we also received DAA failure patients from 32 TCs and 2 model treatment centers (MTCs).

Patients at high risk of reinfection after DAA treatment failure were excluded from the study population. This exclusion applied to individuals on hemodialysis and those with hemoglobinopathies, as these patients frequently require transfusions of blood and blood products. Additionally, we excluded all patients who had percutaneous exposure to potential infection sources either during DAA therapy or before achieving SVR12. This included individuals with a history of intravenous drug use, hemodialysis, recent surgery, blood transfusions, or high-risk sexual behavior. Patients who were noncompliant or received inadequate doses of DAA were also excluded from the study population.

### 2.2. Assessment

Upon enrollment, demography and anthropometry data were meticulously recorded. This included details such as age, gender, body weight, and other relevant demographic information. Furthermore, the study documented the participants' previous treatment regimens including duration and adherence to therapy. From the database, all essential laboratory investigations including complete blood count (CBC), liver function tests (LFTs), and renal function tests (RFTs) were recorded. Additional information comprised HCV RNA levels and abdominal ultrasonography. Genotyping was restricted only in patients who needed a retreatment regimen. Before retreatment, fibrosis was assessed by transient elastography, APRI, and FIB4.

#### 2.2.1. Viral Load Estimation and Genotyping

HCV RNA concentrations in serum or EDTA plasma were quantified using the COBAS AmpliPrep/COBAS TaqMan HCV Quantitative test (Version 2.0; Thermo Fisher Scientific, Waltham, MA, United States; lower limit of quantification 15 IU/mL) or the Abbott m2000 system (Abbott, Chicago, IL, United States; lower limit of quantification 12 IU/mL). HCV genotype was determined by the VERSANT HCV Genotype INNO-LiPA Assay V2.0 (Siemens Healthcare Diagnostics, Tarrytown, NY, United States).

#### 2.2.2. Liver Stiffness Measurement (LSM)

Liver stiffness was measured by transient elastography (FibroScan, Echosens, Paris, France) using a medium probe. LSM was performed by the study investigators. Ten valid measurements were obtained and reported as a median value in kilopascal (kPa). Only measurements with at least 10 valid acquisitions, a success rate > 60%, and an interquartile range over median (IQR/M) value < 30% were included in the study. The cutoffs used were > 12.5 kPa for cirrhosis and > 8.6 kPa for advanced fibrosis [11].

### 2.3. Retreatment Regimen

At present, NVHCP provides only first-line DAA. Ribavirin is available only in the ATC. All patients received SOF/VEL (400/100 mg) fixed-dose combination with weight-based ribavirin for 24 weeks.

### 2.4. End Point

The primary endpoint was the proportion of DAA failure patients who did not achieve SVR12 with the retreatment regimen based upon the combination of SOF/VEL with ribavirin.

### 2.5. Statistical Analysis

All data were entered into a Microsoft Excel Spreadsheet. Data analysis was carried out using appropriate statistical software using SPSS 28-2021 version. The results were expressed as percentage, median (IQR).

## 3. Result

### 3.1. Patient Population

**Figure 1:**
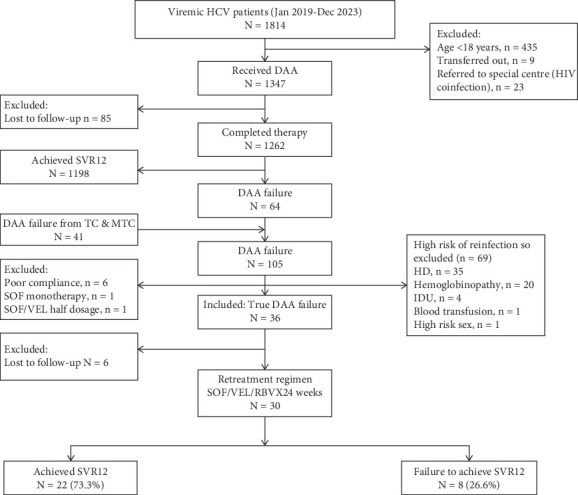
CONSORT flow diagram. Flow of participant in the study.

Between January 2019 and December 2023, our center registered 1814 HCV-infected patients with detectable viremia, of whom 1347 patients were offered DAA. Of these, 1262 patients completed therapy. One thousand one hundred ninety-eight (94.9%) patients achieved SVR12, and 68 patients (5.1%) failed to achieve SVR12 (Figure 1). In addition, we received 41 patients who had experienced treatment failure from other TCs or MTCs. After excluding 69 patients, we included 36 patients in our study; however, six patients could not be tracked for further evaluation. Therefore, the study included 30 patients who were offered a retreatment regimen (Figure 1). Detailed patient characteristics are provided in Table 1. Most patients were male (64.5%), with a median age of 45 years. All patients received treatment according to NVHCP guidelines [4]. Among the study cohort, 25 patients were noncirrhotic, 4 had compensated cirrhosis, and 1 had decompensated cirrhosis (Table 1). The median viral load before first-line treatment was 324,115 IU/mL (IQR: 20,464–1,454,988), and before retreatment, it was 87,882 IU/mL (IQR: 9870–484,909). The genotype distribution was as follows: Genotype 3a, 63.3% (*n* = 19); Genotype 3b, 33.3% (*n* = 10); and Genotype 1a, 3.3% (*n* = 1) (Table 1).

### 3.2. Efficacy of Retreatment Regimen

All patients received a retreatment regimen based on a fixed-dose combination of SOF/VEL and weight-based ribavirin for 24 weeks. Twenty-two patients (73.3%) achieved SVR12, while 8 (26.6%) did not achieve SVR12 (Figure 1). No major adverse events were reported. Of the eight patients who failed to achieve SVR12, five were noncirrhotic, and three had cirrhosis (Table 1). The median pre-retreatment HCV RNA for treatment failures was 1,422,070 IU/mL (IQR: 361,916–6,515,695) (Table 2). Detailed characteristics of individual patients who did not achieve SVR with the retreatment regimen are summarized in Table 2. As NVHCP does not provide third-line therapy, no patients received further treatment.

## 4. Discussion

Chronic viral hepatitis is a significant public health issue [3]. To reduce its associated mortality and morbidity, many countries have launched national programs to address viral hepatitis. The government of India has introduced its own national program, providing drugs and diagnostic services free of cost [4].

DAAs have real-world efficacy rates of 90%–98%, yet some patients do not achieve a SVR. Currently, the NVHCP does not define a standard retreatment regimen, offers a limited selection of drugs, and does not require genotyping before treatment. Patients who experience treatment failure are referred to apex centers for advanced care.

To improve the program's effectiveness, it is crucial to understand the rate of treatment failure and identify optimal retreatment regimens. In our single-center retrospective study, we found an overall virological failure rate of 5.0%. A large-scale study from India by Dhiman et al. reported that virological failure is 8% [5]. The combination of voxilaprevir, velpatasvir, and sofosbuvir (VOX/VEL/SOF) is the preferred second-line treatment for patients with prior DAA failures, achieving viral eradication rates above 95% [12]. However, VOX/VEL/SOF is currently unavailable in the NVHCP, which limits retreatment options. Data on treatment failures within the national program also remain limited.

Given the ethical imperative to provide retreatment, we included 30 patients with prior treatment failure and treated them with a 24-week regimen of SOF/VEL/RBV. Seventy-three percent of these patients achieved SVR12, which is consistent with the findings of a study by Amit et al., which reported an SVR12 rate of 80% for the same regimen [10]. Another retrospective study demonstrated an SVR12 rate of 100% with a SOF/VEL-based regimen, with or without ribavirin [13]. So our study reflects similar trends, though our efficacy rate is slightly lower.

Our findings underscore the potential benefit of the SOF/VEL/RBV regimen for optimizing treatment outcomes.

## 5. Limitations

This study has several limitations. Being a retrospective, single-center analysis, the findings may not be fully representative of outcomes under the NVHCP. Although the NVHCP recommends the use of SOF/VEL for patients with compensated cirrhosis, one such patient in our cohort received SOF/DCV as first-line therapy. Additionally, while patients with prior treatment failure from other centers were included, SVR12 data from those centers were not available. The small number of treatment failure cases limited the ability to perform meaningful subgroup analyses or identify predictors of nonresponse. Furthermore, the study did not evaluate disease progression or long-term outcomes among patients who failed the initial treatment. Although no adverse events were reported and the retreatment regimen appeared well tolerated, the retrospective design may have contributed to underreporting of adverse effects. Lastly, as the majority of patients had Genotype 3 HCV, the generalizability of these findings to other genotypes is limited.

## 6. Conclusion

This single-center retrospective study under NVHCP has shown that DAAs are highly effective in real-life scenarios. The retreatment regimen with sofosbuvir, velpatasvir, and ribavirin for 24 weeks achieved a SVR12 in 73% of cases. This regimen may be considered a second-line option. In the future, the program should develop a management protocol for patients with DAA failure, including the availability of the voxilaprevir, sofosbuvir, and velpatasvir regimen.

## Figures and Tables

**Table 1:** Baseline demographic and clinical characteristic of treatment failure patients.

	**Total patients (** **n** = 30**)**
Age	45 (19–68)
Sex (male)	20 (64.5%)
Body weight (kg)	56 (50.7–65.7)
*Laboratory parameters*	
HB (g/dL)	10.0 (9–11.3)
Platelet	175 (160–238)
Serum total bilirubin (mg/dL)	0.97 (0.73–1.5)
ALT (IU/L)	45 (29–79.7)
AST (IU/L)	47 (32.5–94.7)
Albumin (g/dL)	3.7 (2.9–4.3)
Creatinine (mg/dL)	0.98 (0.79–1.1)
*Noninvasive markers of fibrosis*	
APRI	0.65 (0.40–0.90)
FIB4	1.42 (0.88–2.21)
LSM	7.35 (5.0–8.3)
*Viral load*	
HCV RNA in treatment naïve patients (IU/mL)	324,115 (20,464–1,454,988)
HCV RNA before retreatment (IU/mL)	87,882 (9870–484,902)
*Genotype*	
G3a	19 (63.3)
G3b	10 (33.3)
G1a	1 (3.03)
*Disease phenotype*	
Noncirrhotic	25 (83.3)
Compensated cirrhotic	4 (13.3)
Decompensated cirrhosis	1 (3.3)
*First-line regimen*	
SOF + daclatasvir ×12 weeks	27 (90)
SOF + velpatasvir ×12 weeks	2 (6.6)
SOF + velpatasvir ×24 weeks	1 (3.3)

*Note:* Data are expressed as median and IQR or number (%) unless otherwise specified.

**Table 2:** Characteristics of patients who did not respond to retreatment regimen.

**Serial no**	**Age**	**Sex**	**APRI**	**FIB4**	**LSM**	**Genotype**	**Disease phenotype**	**Viral load before retreatment (IU/mL)**	**Previous treatment**
1	49	M	0.9	2.89	44.1	G3a	Decompensated cirrhosis	853,726	SOF/VEL ×24 week
2	24	M	0.9	2.56	5.1	G3a	Noncirrhotic	123,121	SOF/DCV ×12 week
3	38	F	0.5	0.51	4.5	G1b	Noncirrhotic	600,711	SOF/DCV ×12 week
4	28	M	3.3	0.88	26.8	G3b	Compensated cirrhotic	1,990,414	SOF/DCV ×24 week
5	46	M	1.6	0.85	5.0	G3a	Noncirrhotic	7,834,555	SOF/DCV ×12 week
6	68	M	0.7	1.76	22.3	G3a	Compensated cirrhotic	7890	SOF/DCV ×12 week
7	58	M	0.4	0.89	5.3	G3b	Noncirrhotic	5,581,359	SOF/DCV ×12 week
8	34	F	0.3	0.80	7.4	G3a	Noncirrhotic	7,450,031	SOF/DCV ×12 week

## Data Availability

The data that support the findings of this study are available from the corresponding author upon reasonable request.

## Nomenclature

NVHCP: National Viral Hepatitis Control Program
TC: treatment center
MTC: model treatment center
ATC: apex treatment center
TE: transient elastography
kPa: kilopascal
LS: liver stiffness
SVR12: sustained virological Response 12
DAA: direct-acting antivirals
APRI score: aspartate aminotransferase (AST)-to-platelet ratio index score
FIB4 score: Fibrosis-4 score
WHO: World Health Organization
IDU: IV drug user
SOF: sofosbuvir
VEL: velpatasvir
VOX: voxilaprevir
DCV: daclatasvir
RBV: ribavirin
